# Missing magnetism in Sr_4_Ru_3_O_10_: Indication for Antisymmetric Exchange Interaction

**DOI:** 10.1038/s41598-017-03648-2

**Published:** 2017-06-20

**Authors:** Franziska Weickert, Leonardo Civale, Boris Maiorov, Marcelo Jaime, Myron B. Salamon, Emanuela Carleschi, André M. Strydom, Rosalba Fittipaldi, Veronica Granata, Antonio Vecchione

**Affiliations:** 1 Los Alamos National Laboratory, MPA-CMMS, Los Alamos, NM 87545 USA; 2 Florida State University, NHMFL, Tallahassee, FL 32310 USA; 3 Department of Physics, University of Texas at Dallas, Richardson, TX 75080 Dallas, USA; 4 Department of Physics, University of Johannesburg, Auckland Park, 2006 South Africa; 5 CNR-SPIN Institute Sede Secondaria di Salerno and University of Salerno, Via Giovanni Paolo II, I-84084 Fisciano, Italy

## Abstract

Metamagnetism occuring inside a ferromagnetic phase is peculiar. Therefore, Sr_4_Ru_3_O_10_, a *T*_*C*_ = 105 K ferromagnet, has attracted much attention in recent years, because it develops a pronounced metamagnetic anomaly below *T*_*C*_ for magnetic fields applied in the crystallographic *ab*-plane. The metamagnetic transition moves to higher fields for lower temperatures and splits into a double anomaly at critical fields *H*_c1_ = 2.3 T and *H*_c2_ = 2.8 T, respectively. Here, we report a detailed study of the different components of the magnetization vector as a function of temperature, applied magnetic field, and varying angle in Sr_4_Ru_3_O_10_. We discover for the first time a reduction of the magnetic moment in the plane of rotation at the metamagnetic transition. The anomaly shifts to higher fields by rotating the field from *H* ⊥ *c* to *H* || *c*. We compare our experimental findings with numerical simulations based on spin reorientation models taking into account magnetocrystalline anisotropy, Zeeman effect and antisymmetric exchange interactions. While Magnetocrystalline anisotropy combined with a Zeeman term are sufficient to explain a metamagnetic transition in Sr_4_Ru_3_O_10_, a Dzyaloshinskii-Moriya term is crucial to account for the reduction of the magnetic moment as observed in the experiments.

## Introduction

Sr_4_Ru_3_O_10_ belongs to the Ruddlesden-Popper family of ruthenium oxide perovskites Sr_*n*+1_Ru_*n*_O_3*n*+1_. This class of metallic compounds caught much attention in recent years due to its rich variety of ground states. Sr_2_RuO_4_ the *n* = 1 member, is discussed as an example of rare *p*-wave superconductivity^1^. A quantum critical endpoint covered by a high entropy phase was found in the *n* = 2 layer system Sr_3_Ru_2_O_7_^2^. The compound Sr_4_Ru_3_O_10_ (*n* = 3) discussed here shows ferromagnetism below *T*_*C*_ = 105 *K* ^3^. Neutron diffraction experiments in zero magnetic field reveal ordering of the Ru moments along the *c*-axis. No ferromagnetic (FM) or antiferromagnetic (AFM) correlations are observed in the *ab*-plane^4, 5^. Sr_4_Ru_3_O_10_ contains four inequivalent Ru sites with two different magnetic moments of 0.9 *μ*_*B*_ and 1.5 *μ*_*B*_ sitting on outer and inner RuO layers, respectively. The magnetic unit cell contains 8 of the smaller and 4 of the larger magnetic moments averaging to 1.1 *μ*_*B*_ per Ru. The higher order ruthenate SrRuO_3_ with *n* = ∞ also orders FM at a Curie temperature of 165 K^6, 7^. The Sr_*n*+1_Ru_*n*_O_3*n*+1_ are strongly 2-dimensional electron systems with the trend to become more isotropic for higher *n*, because of their layered structure. Two-dimensionality is reflected in anisotropic transport properties as seen for Sr_4_Ru_3_O_10_ in the ratio of the electrical resistivity ${\rho }_{c}/{\rho }_{ab}\simeq 400$ ^8^ and confirmed by optical conductivity experiments^9^.

Metamagnetism is a phenomenon observed in magnetic materials, where hidden magnetism is suddenly uncovered by the application of an external field. The origin of metamagnetism can be spin flip transitions in antiferromagnets^10–12^, but also changes of the band structure in itinerant electron systems. Latter scenarios are in the vast majority described on the basis of the well-known Stoner model^13^ and refined^14–16^, to accommodate special cases, e.g. Fermi surface reconstruction^17, 18^ or in the vicinity of a quantum critical point^19, 20^. In a general description, metamagnetism is a phase transition or crossover from a magnetically disordered or ordered state with small net magnetization to a field polarized (FP) or partially FP state. In the case of Sr_4_Ru_3_O_10_ the term metamagnetism refers to the sudden increase in the magnetization when the field applied in the (*ab*) in-plane of this layered compound exceeds 2 T. Magnetism is hidden only because the spontaneous moment is mainly aligned with the easy *c*-axis at smaller fields; nonetheless, we continue to refer to this as metamagnetic (MM) transition. Interestingly, the saturation magnetization above the MM transition (*H* ⊥ *c*) is about 10% smaller than the saturated moment for *H* || *c* ^3, 21^, which points to a more complex underlying scenario for the metamagnetism than just a simple spin flip transition. While the MM transition in Sr_4_Ru_3_O_10_ was discovered from early on in flux grown single crystals^3, 21^, it took more than a decade to improve the crystal quality to a level to see a double step in the magnetization at the MM transition^22^. This strong dependence of physical properties on the crystal purity is a characteristic signature of strontium ruthenates Sr_*n*+1_Ru_*n*_O_3*n*+1_ and was also observed in the sister compound Sr_3_Ru_2_O_7_^2, 23–25^. The MM transition in Sr_4_Ru_3_O_10_ develops below 68 K as a double-transition close to zero field and shifts gradually to about 2.5 T with temperatures down to 1.7 K^22^. Carleschi *et al*.^22^ speculate that the double transition originates either in the ordering of Ru magnetic moments on two inequivalent crystallographic sites or in the presence of two van Hove singularities in the density of states close to the Fermi level. A transport study based on electrical resistivity^26, 27^ reveals steps in the magnetoresistance at various critical fields around *H*_*c*_ accompanied by pronounced hysteresis. Fobes *et al*.^27^ interpret the transport data as domain movement of regions with high and low electronic spin polarization. Anomalous behavior at the MM transition was also observed in specific heat experiments^28^ up to 9 T and in thermopower investigations^29^. Neutron diffraction experiments up to 6 T reveal a change of lattice parameters at the critical field *H*_*c*_^4^. Field and pressure dependent Raman measurements^30^ as well as a recent study of thermal expansion and magnetostriction^31^ confirm strong magnetoelastic coupling in Sr_4_Ru_3_O_10_.

This work aims to increase our understanding of MM phenomena in 4*d* oxides in general and the peculiar MM transition inside the ferromagnetic order of Sr_4_Ru_3_O_10_ in particular. We carry out magnetization measurements up to 7 T and down to lowest temperatures of 0.46 K and under rotational fields between the *c*-axis and the *ab*-plane as well as (*ab*) in-plane rotation. Our investigations include a detailed analysis of the behavior of the magnetization modulus *M* at the MM transition and its individual components *M*_*ab*_ and *M*_*c*_ simultaneously. In the following, we analyze and interpret our data in a localized picture, meaning the magnetic moments are mainly confined on the Ru^4+^ sites in the crystal structure of Sr_4_Ru_3_O_10_. This scenario is supported by neutron diffraction experiments which have determined the spin and orbital momentum distribution in great detail^4, 5^. Our main discovery is the observation of a reduced measured moment at the MM transition caused by a spin component pointing out of the rotational plane which we assert can best be explained by significant anisotropic exchange interactions in Sr_4_Ru_3_O_10_.

## Results

**Figure 1:**
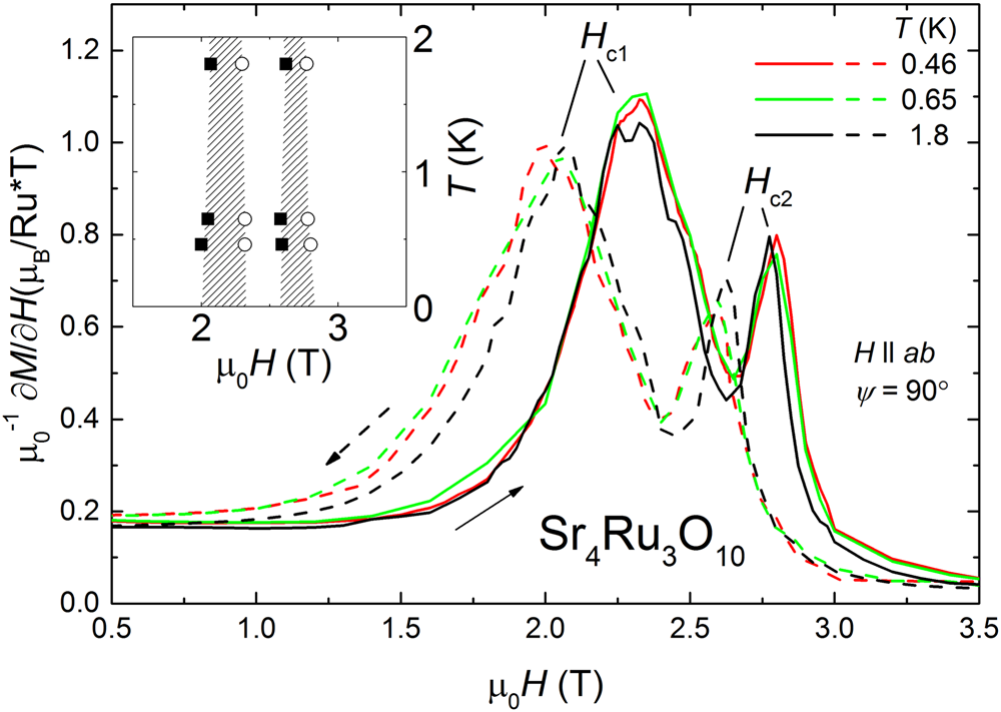
Field derivative of the magnetization $\partial M/\partial H$ at 1.8 K, 0.65 K and 0.46 K with clear anomalies at two critical fields *H*_*c*1,2_. Solid lines show measurements during increasing field sweeps and dashed lines during decreasing field sweeps as labeled by arrows. The inset shows *H* − *T* phase diagram close to the MM transition with regions of hysteresis marked as striped patterns.

At first, we focus on the magnetization measured for *H* ⊥ *c* at temperatures below 2 K. Figure 1 shows the susceptibility $\partial M/\partial H$ between 0.5 T and 3.5 T. We observe a clear double MM phase transition with a main anomaly at *H*_*c*1_ = 2.3 T and a second anomaly at *H*_*c*2_ = 2.8 T for increasing field as observed by Carleschi *et al*.^22^. Our new experimental data down to 0.46 K clarify that the transition neither sharpens to lower temperatures nor is there splitting into more distinct anomalies. The inset in Fig. 1 shows the *H* − *T* phase diagram with near-vertical phase boundaries for *T* → 0 at the MM transition. Both anomalies are shifted by −0.3 T for measurements in decreasing magnetic field. The size of the hysteretic region, marked as striped pattern, remains similar for all temperatures below 1.8 K.

**Figure 2:**
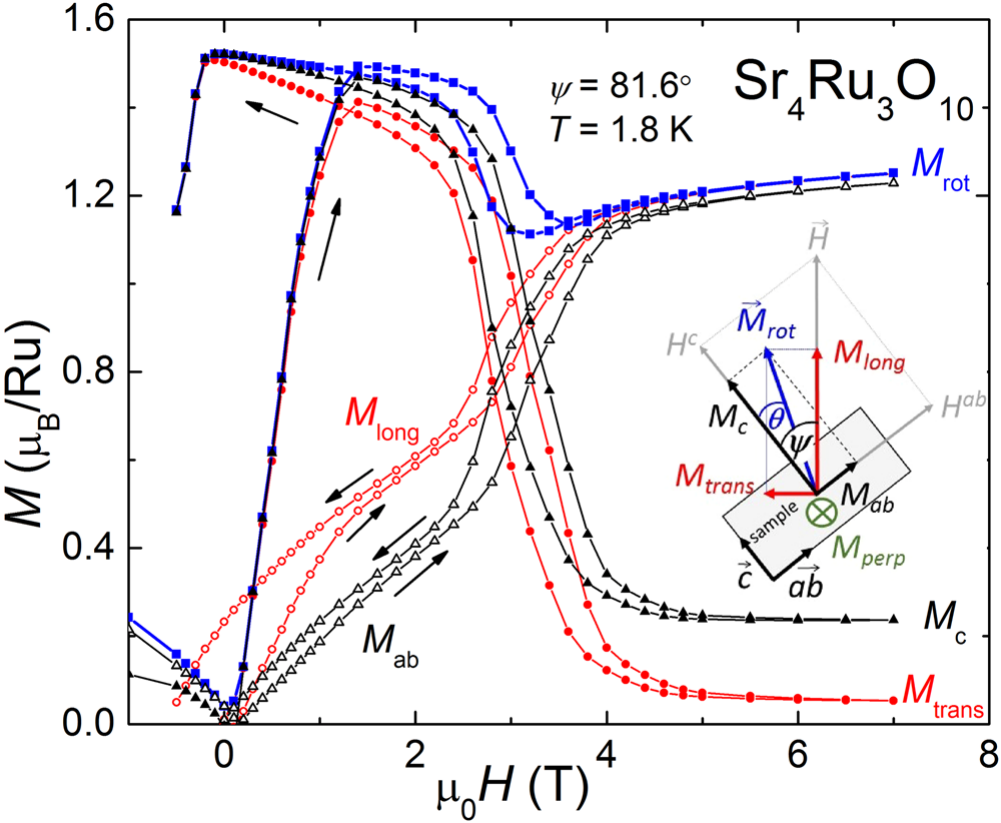
The inset shows a geometrical sketch of the sample Sr_4_Ru_3_O_10_ mounted inside the SQUID magnetometer. *ψ* is the rotation angle of the applied field $\vec{H}$ and *θ* the angle of the magnetization $\vec{M}$, both in respect to the magnetic easy *c*-axis. *M*_*long*_ and *M*_*trans*_ are measured components of $\vec{M}$ parallel and perpendicular to the applied field in the plane of rotation. The component *M*_*perp*_ parallel to the axis of rotation is not captured during the measurement. The main panels compares the different components of the magnetization *M*_*long*_, *M*_*trans*_, *M*_*ab*_, *M*_*c*_, and the modulus *M*_*rot*_ versus magnetic field *H* measured at 1.8 K for *ψ* = 81.6°.

The operation mode of the SQUID magnetometer allows the simultaneous collection of longitudinal *M*_*long*_ and transversal component *M*_*trans*_ of the sample magnetization in respect to the applied magnetic field $\vec{H}$ as sketched in the inset of Fig. 2 (see Methods). Any magnetization component *M*_*perp*_ occurring perpendicular to the rotational plane is not recorded during the measurements. This geometry allows us to calculate the magnetization ${M}_{rot}^{2}={M}_{long}^{2}+{M}_{trans}^{2}$ in the rotational plane. The knowledge of the rotation angle $\psi$ and relation $\tan (\psi -\theta )={M}_{trans}/{M}_{long}$ enables the determination of the angle of the magnetization *θ* with respect to the magnetic easy axis *c* in the rotational plane. We can now calculate *M*_*ab*_ = *M*_*rot*_ sin *θ* and *M*_*c*_ = *M*_*rot*_ cos *θ*, the magnetization occurring in the plane of rotation. Note, we follow closely the notation of angles used for magnetic anisotropic materials. Figure 2 illustrates the different components of the magnetization for one particular measurement with *ψ* = 81.6° taken at 1.8 K. Prominent feature is the hysteresis loop around ±1 T, caused by FM domain dynamics. The longitudinal magnetization *M*_*long*_ increases moderately in small fields and shows a sudden rise at *H*_*c*1,2_ $\simeq$ 3 T at the MM transition. *M*_*trans*_ on the other hand consists mainly of the *M*_*c*_ component with a sudden decrease of the magnetization at the same critical fields *H*_*c*1,2_. ${M}_{trans}\ne 0$ above *H*_*c*1,2_ indicates incomplete field polarization meaning that $\vec{M}$ is not perfectly aligned with $\vec{H}$. This observation points to the presence of magnetic anisotropy. The calculated magnetization modulus *M*_*rot*_ in the plane of rotation is depicted as black line in Fig. 2. We find a maximum moment of 1.5 *μ*_*B*_ slightly higher than obtained in neutron experiments^4, 5^, but in good agreement with previous magnetization studies^21, 32^. Most peculiar is that *M*_*rot*_ drops suddenly below 1.2 *μ*_*B*_ at the MM transition and only recovers partially to 1.2 *μ*_*B*_ up to maximum applied field of 7 T. This missing component of the magnetic moment in Sr_4_Ru_3_O_10_ was never recognized before. Furthermore, we observe strong hysteresis at the MM transition between up and down measurements as reported in previous investigations^8, 21, 22, 27, 32^.

**Figure 3:**
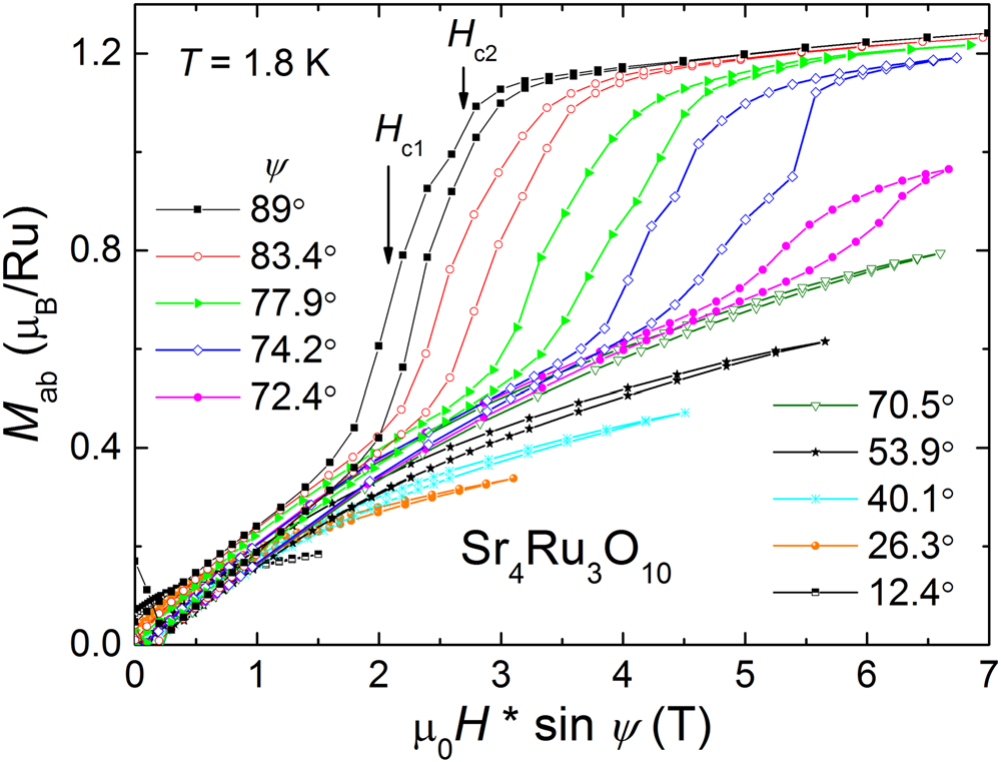
*ab*-plane magnetization *M*_*ab*_ as a function of *H* ^*ab*^ = *H* sin *ψ* is shown for selected angles *ψ* between 89° and 12.6° measured at 1.8 K. A clear double step is observed at *H*_*c*1,2_ as labeled by arrows for *ψ* = 89°.

Geometrical effects can distort magnetic properties during magnetization experiments. To avoid this problem, we plot in Fig. 3 *M*_*ab*_ as a function of the field component in the *ab*-plane ${H}^{ab}=H\,\sin \,\psi$ to examine how *H*_*c*1,2_ change with *ψ*. In contrast to previous results by Jo *et al*.^33^ obtained by torque magnetometry, we observe a clear simultaneous increase of both critical fields *H*_*c*1,2_ to higher values while rotating from *H* ⊥ *c* to *H* || *c*. In fact, *H*_*c*1,2_ move out of the observable field range of *H* ≤ 7 T for *ψ* ≲ 72°. A similar shift to higher critical fields was observed in measurements of the longitudinal magnetoresistance for currents *j* || *c* and *j* ⊥ *c* as a function of rotating magnetic field as reported by Fobes *et al*.^8, 27^.

**Figure 4:**
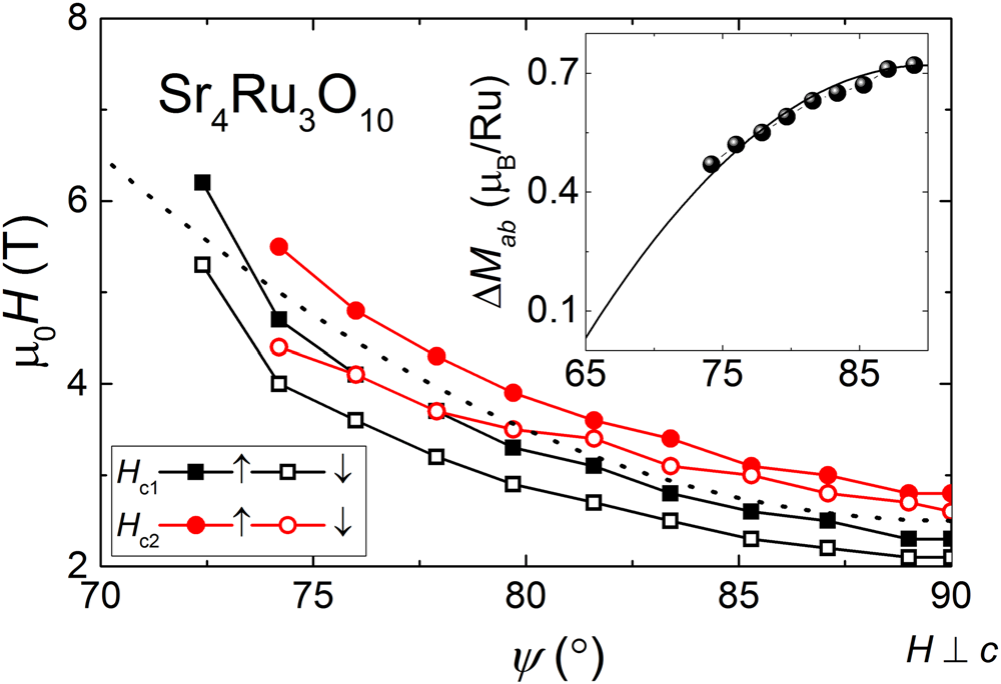
Angular *ψ* dependent shift of the double anomaly at the MM transition in Sr_4_Ru_3_O_10_. Solid points mark positions for increasing and empty points decreasing field sweeps. The dotted line is a quadratic fit to the data. The inset shows the reduction of the magnetization step Δ*M*_*ab*_ at the MM transition as a function of *ψ* including a quadratic extrapolation marked as solid line.

**Figure 5:**
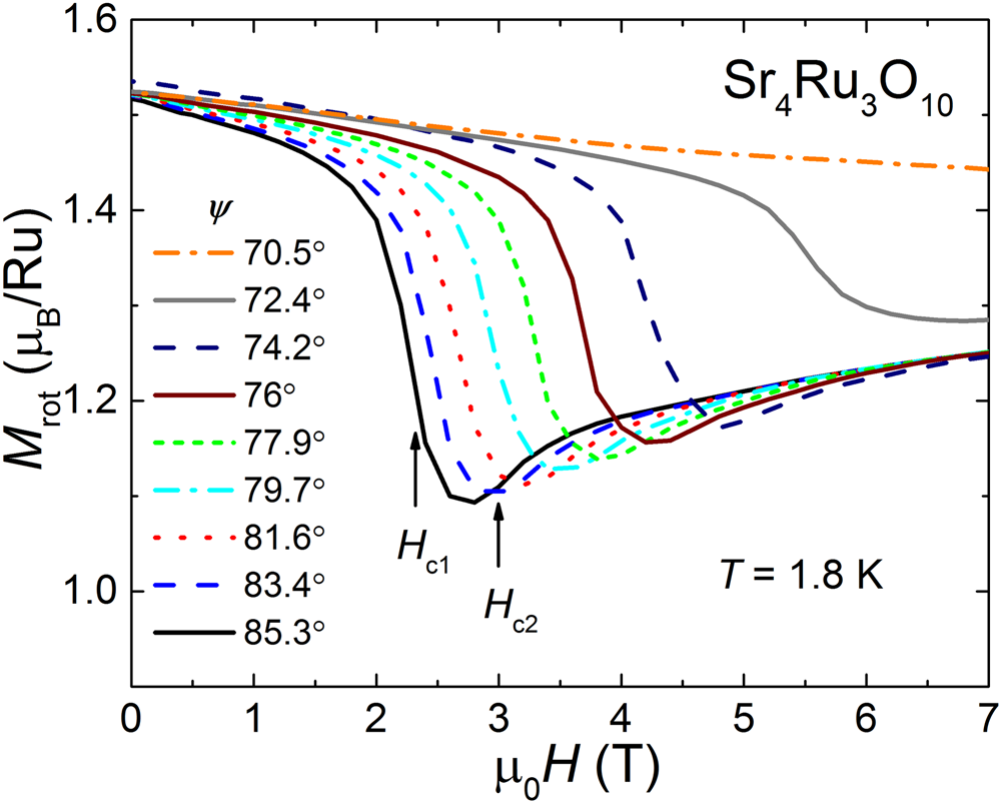
Magnetization modulus *M*_*rot*_ in the rotational plane versus magnetic field *H* for angles *ψ* between 85.3° and 70.5° in decreasing fields. The drop in *M*_*rot*_ coincides with *H*_*c*1_ and the step-like increase with *H*_*c*2_ as marked by arrows for the measurement at *ψ* = 85.3°.

Figure 4 summarizes the critical fields *H*_*c*1,2_ in the *H* − *ψ* phase diagram for field up and down sweep measurements. The difference *H*_*c*1_ − *H*_*c*2_ increases slightly with smaller *ψ*. As mentioned above, the double anomaly is accompanied by significant hysteresis. The inset of Fig. 4 shows the evolution of combined step size Δ*M*_*ab*_ of both MM transitions for decreasing *ψ* which were extracted from the curves in Fig. 3. It follows a quadratic fit function marked as solid line and extrapolates to zero step size at about 65°. The magnetization modulus *M*_*rot*_ recorded in the plane of rotation for *ψ* between 85.3° and 70.5° is plotted in Fig. 5. We only show field-down sweep measurements for clarity. Striking is the occurrence of a drop from about 1.5 *μ*_*B*_ to below 1.2 *μ*_*B*_ at the critical field of the main anomaly *H*_*c*1_ followed by a minimum and a small step at the second anomaly at *H*_*c*2_. The described features are marked in Fig. 5 by arrows for the measurement at *ψ* = 85.3°. The MM anomaly broadens and moves to higher fields for decreasing angles *ψ*.

So far, we only carried out magnetization measurements under rotational fields at 1.8 K and did not expand to higher temperatures. Therefore, we cannot conclude with certainty, how the “moment loss” changes with increasing temperature. We have shown, however, that *H*_*c*1_ and *H*_*c*2_ of the double step in *M*_*ab*_ are intimately connected to the missing moment and represent basically the same critical magnetic fields, where the magnetization drops and partially recovers. Please compare Figs 3 and 5. Carleschi *et al*. found out in a temperature study^22^ that the double transition occurs right below the ferromagnetic ordering temperature *T*_*C*_ = 105 K. This observation makes us believe that the effect of a missing moment occurs right below *T*_*C*_ as well with some temperature broadening.

## Discussion

The “loss” of magnetic moment in the rotational plane can be explained either by partial AFM alignment or by a moment *M*_*perp*_ occurring perpendicular to the rotation (parallel to rotation axis of *ψ*). The first scenario can be excluded based on neutron experiments where no short or long range AFM coupling neither in zero nor in magnetic fields *H* > *H*_*c*1,2_ was observed in the *ab*-plane^4, 5^. The second scenario is rather unexpected since magnetic moments tend to align with field and stay within the rotational plane, if no further coupling is present. We want to focus in our discussion on two mechanisms that potentially lead to a *M*_*perp*_ component in the magnetization. First one is based on general magnetocrystalline anisotropy in tetragonal symmetry, with an easy *c*-axis and 4-fold in-plane anisotropy. The second mechanism is antisymmetric exchange between spins, also called Dzyaloshinskii-Moriya (DM) interaction, causing a canting of the spins ${\vec{S}}_{i}\times {\vec{S}}_{j}$.

We have to have a closer look at the crystal structure of Sr_4_Ru_3_O_10_ in order to understand and model its magnetic anisotropy caused by spin-orbit coupling. Sr_4_Ru_3_O_10_ crystals consist of three layers of corner sharing RuO_6_ octahedra separated by a double layer of Sr-O. Primary Bragg reflections in synchrotron experiments can be indexed assuming a tetragonal unit cell with space-group *I*4/*mmm*, but a more detailed analysis of secondary reflections reveals orthorhombic *Pbam* symmetry^3^. The lower symmetry originates in *c*-axis rotation of the RuO_6_ octahedra that are correlated between different layers, meaning +11.2° clockwise rotation for inner and −5.6° counterclockwise rotation for outer layers.

The free energy *F* accounting for magnetocrystalline anisotropy in a tetragonal lattice, can be modeled^34^ by

1 $$F={F}_{0}+{K}_{1}\,{\sin }^{2}\,\theta +({K}_{2}+{K}_{3}\,\sin \,\mathrm{(2}\phi ))\,{\sin }^{4}\,\theta -{F}_{Z}.$$

*F*_0_ is a constant background contribution independent of $\vec{H}$ or $\vec{M}$. $\vec{M}$ is expressed in polar coordinates (*θ*, *φ*), with *θ* = 0 along the crystal *c*-axis and *φ* = 0 defining the in-plane hard axis for *K*_3_ < 0. Here, *K*_1_ > 0 defines the easy direction and the Zeeman term *F*_*Z*_ can be written as

2 $${F}_{Z}=MH(\sin \,\theta \,\sin \,\psi (\cos \,\omega \,\cos \,\phi +\,\sin \,\omega \,\sin \,\phi )+\,\cos \,\theta \,\cos \,\psi ).$$

The applied field has the spherical coordinates (*ψ*, *ω*), with *ω* = 0 corresponding to field rotation from the *c*-axis to the in-plane hard direction and *ω* = *π*/4, field rotation to the easy direction. We used numerical minimization of equation (1) to determine *θ* and *φ* as functions of the applied field. In the uniaxial case with *K*_3_ = 0, the MM behavior in *M*_*ab*_ and *M*_*c*_ at the critical field *H*_*c*_ is reproduced by choosing correct parameters *K*_1_ and *K*_2_ (data not shown). However, uniaxial anisotropy is unable to reproduce any reduction of the magnetization in *M*_*rot*_, since the moment always stays in the rotational plane. Therefore, we considered in-plane anisotropy with ${K}_{3}\ne 0$ in the next step.

Note, we do not know precisely the in-plane orientation of our sample. However, the rectangular shape suggests that *ψ* rotation axis is parallel to one of the principal axes such as [100] or [110]. For 4-fold tetragonal symmetry either one of them would be the intermediate or hard axis, respectively. We consider in the following a projection of $\vec{H}$ onto the magnetic hard axis in the *ab*-plane with *ω* = 0, because tilting of $\vec{M}$ towards the hard axis forces the magnetic moments to align spontaneously toward either one of the intermediate axes, which are 45° apart from the hard axis. This spontaneous alignment ±45° is energetically degenerated and could lead to domain formation with an overall smaller net magnetization as observed in our measurements.

**Figure 6:**
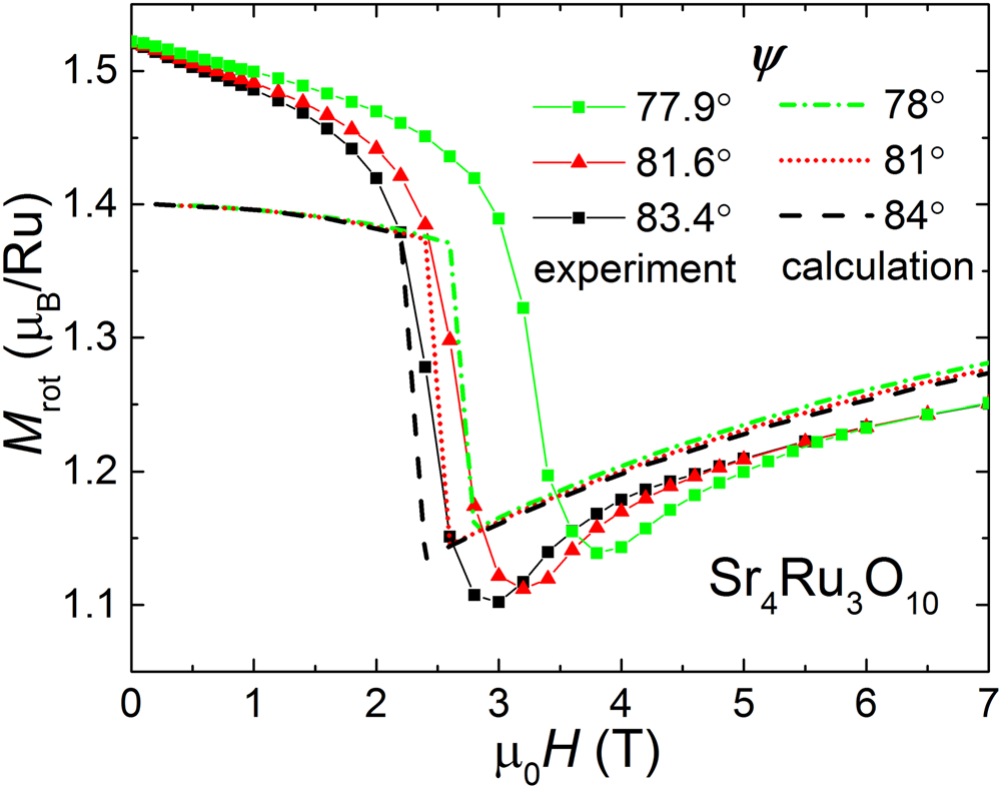
The experimental magnetization moduli *M*_*rot*_ for 3 different angles *ψ* (symbols) measured at 1.8 K in decreasing magnetic field are compared with numerical simulations (broken lines) of the general anisotropy model as described by Eq. (1).

Figure 6 compares numerical results of *ψ* = 78°, 81°, 84° based on equation (1) with experimental data of the magnetization modulus *M* for *ψ* = 77.9°, 81.6°, 83.4°. We are able to reproduce i) a critical field value of about 2.5 T that increases with smaller *ψ*, ii) a drop Δ*M* at *H*_*c*1,2_ that is comparable in size with the experimental data, and iii) a gradual slope *M*(*H*) for *H* > *H*_*c*1,2_. The double feature at the MM transition is missing due to the simplicity of the model. We obtain anisotropy parameters *K*_1_ = 3.1 K, *K*_2_ = 0.1 K and *K*_3_ = −2.2 K in Kelvin energy scale which convert to the following values 300 kJ/m^3^, 10 kJ/m^3^, and −210 kJ/m^3^, respectively, in units widely used in magnetic anisotropy tables. The 4th order parameter *K*_2_ being more than 10 times smaller than *K*_1_ implies that it is irrelevant for the description of the anisotropy in Sr_4_Ru_3_O_10_. For comparison, the 3*d* FM metal cobalt has anisotropy constants of *K*_1_ = 450 kJ/m^3^ and *K*_2_ = 150 kJ/m^3^, which are of similar size as *K*_1_ in Sr_4_Ru_3_O_10_^35^.

**Figure 7:**
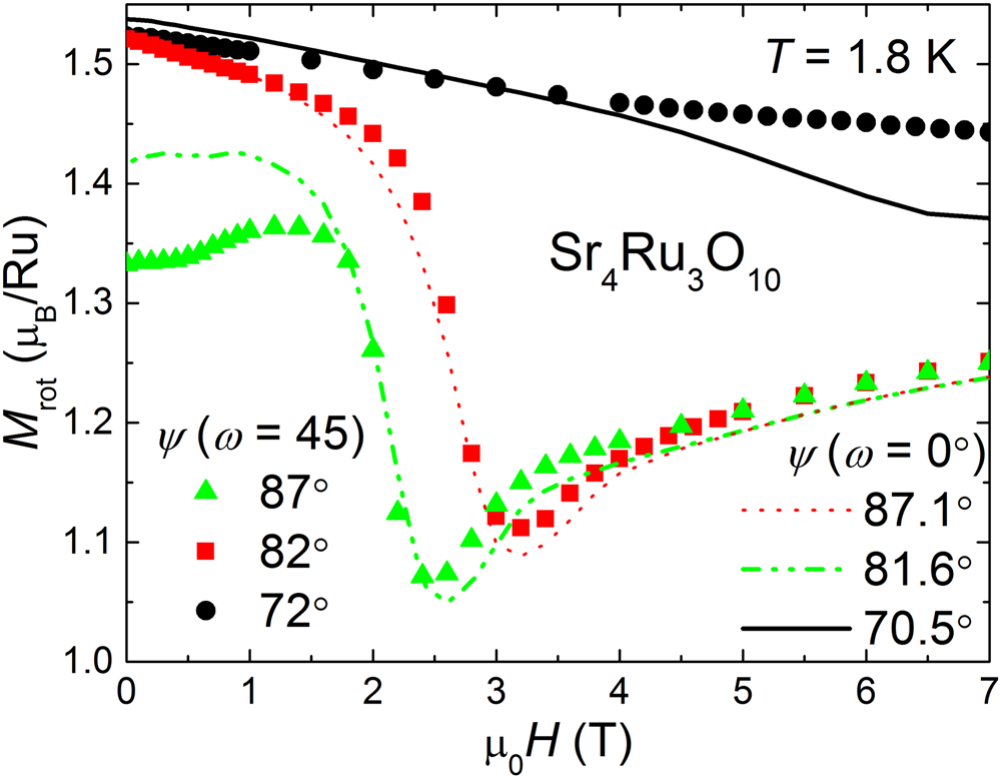
Decreasing field *H* measurements of the magnetization modulus *M*_*rot*_ taken at 1.8 K at three angles *ψ* ≃ 87°, 82°, and 71° are shown for two different in-plane angles *ω* = 0 and 45°. The almost identical results for 0 and 45° disable magnetocrystalline anisotropy being the cause for the loss of magnetic moment in Sr_4_Ru_3_O_10_.

Despite the reasonable agreement between experiment and model, it is necessary to check in a subsequent experiment our initial assumption of tilting the field towards the magnetic hard axis in the *ab*-plane. Therefore, we rotate the sample by *ω* = 45° in the plane and measure again *M* at three different angles *ψ* as shown in Fig. 7. We anticipate the 45° change would bring the intermediate anisotropy axis into the rotation plane. Specifically, the magnetization would rotate toward the intermediate axis with the total magnetization remaining in the rotation plane and therefore no “loss” of magnetic moment effect. Surprisingly, the magnetization *M*_*rot*_ shows exactly the same behavior as for the *ω* = 0 experiments within experimental uncertainty. Even if in both experiments *ω* = 0 and 45°, the plane of *ψ*-rotation would not include exactly the principal axes, we would at least expect the observation of a reduced anomaly in *M*_*rot*_ at *H*_*c*_. Based on our last finding, we exclude general magnetic anisotropy as sole cause for the reduction of moment at the MM transition in Sr_4_Ru_3_O_10_.

**Figure 8:**
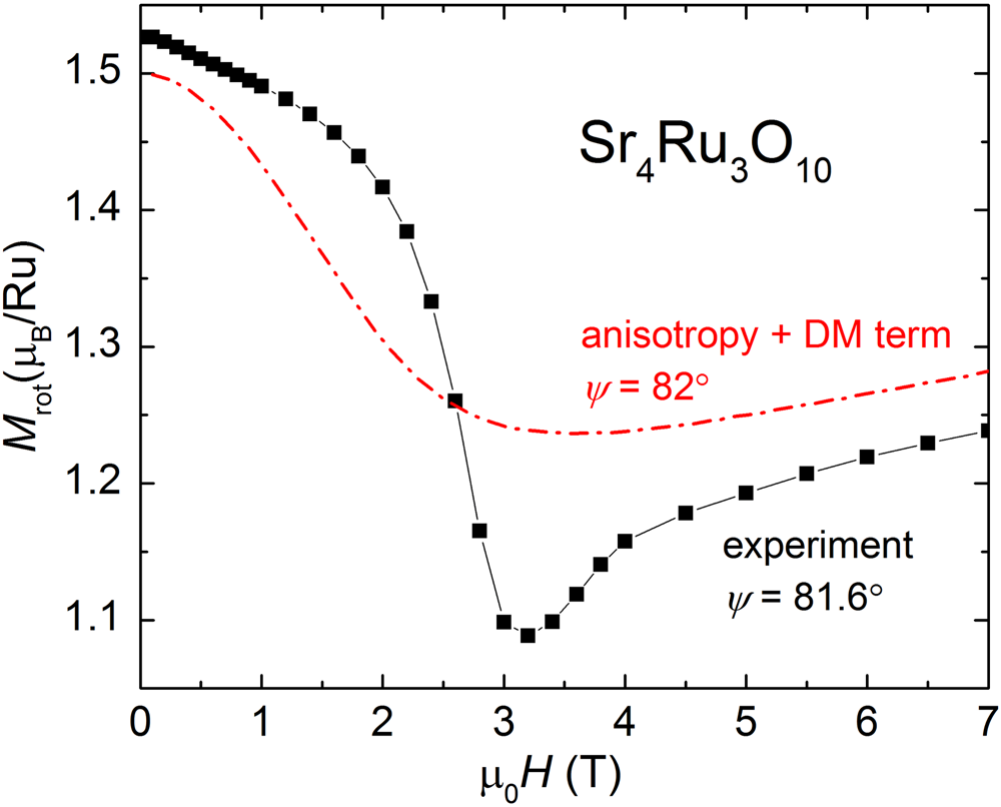
Experimental magnetization modulus *M*_*rot*_ (symbol) in the plane of rotation as a function of magnetic field *μ*_0_ *H* up to 7 T and numerical results including tetragonal magnetocrystalline anisotropy and a DM alike energy term (broken line) for *ψ* ≃ 82°.

Anisotropic exchange interactions caused by spin-orbit coupling under certain symmetry constraints were first considered by *Dzyaloshinsky and Moriya* ^36, 37^ to explain weak FM ordering inside an AFM phase in transition metal oxides. Recently, Bellaiche *et al*.^38^ pointed out that tilting of oxygen octahedra in perovskites can be described by a rotation (pseudo) vector *ν*_*i*_ sitting at position *i* of spin *S*_*i*_. The oxygen octahedra rotation leads to an energy reduction

3 $${\rm{\Delta }}E=K\sum _{i,j}\,({\nu }_{i}-{\nu }_{j})\cdot ({\vec{S}}_{i}\times {\vec{S}}_{j})$$

in analogy to DM antisymmetric exchange coupling. The summation is done over nearest-neighboring spins ${\vec{S}}_{i,j}$ and *K* is a constant. Consequently, we approximate a DM interaction between the in-plane component of the Ru center magnetization and that of the two nearest-neighbor Ru atoms, whose spins are assumed to remain parallel. This gives rise to an effective energy term

4 $${F}_{DM}=-D\,{\sin }^{2}\,(\theta )\,\sin \,\mathrm{(2}\phi )$$

in replacement of the *K*_3_ term of the magnetocrystalline anisotropy. Angle *φ* is interpreted as the angle between in-plane magnetic moments sitting on one inner and two outer layers with *φ* = 0 being the direction of in-plane magnetic field. The change in the dependence on angle *φ* between the magnetization vector and the *c*-axis prevents an accurate minimization of the total energy. Nonetheless, the approximate solution shown in Fig. 8 for *ψ* = 82° does produce a “missing” portion of the total magnetization, with the minimum moving to higher field with decreasing *ψ*. The parameter *D* is approximately 5.3 K and comparable to the energy scale of the magnetocrystalline anisotropy *K*_1_.

We want to point out that the opening of 2*φ* between the inner and outer magnetic moments can be seen as AFM order if it occurs periodically along the *c*-axis. Neutron scattering experiments on periodic holmium-yttrium superlattices^39^ e.g., were able to distinguish between different types of AFM periodicity along[00*l*] with increasing in-plane magnetic field, such as helical, helifan-shaped, fan-shaped and FM order. However, the particular crystal structure of Sr_4_Ru_3_O_10_ with three inequivalent layers of RuO_6_ octahedra connected through a double layer of Sr-O doesn’t give rise to additional periodicity, even if the orientation of the magnetic moments follows a repetitive pattern such as −*φ*, +*φ*, −*φ* along *c* inside the triple layer. As mentioned before, the inner and outer Ru positions are crystallographic inequivalent and carry different magnetic moment sizes. Consistently, in-plane AFM ordering was excluded by neutron scattering studies^4^. Another experimental route to gain insight into the nature of the missing moment is the exploration of transversal magnetoresistance *ρ*_*c*_(*H*, *T*) as a function of temperature along the *c*-direction with in-plane magnetic field (*j* || *c*, *j* ⊥ *H*). In case of a periodic opening 2*φ* of neighboring spins in different layers, we expect to observe coherent scattering and a decreasing magnetoresistance with lower temperature and in higher magnetic field. In case the missing magnetic moment is caused by domain formation of any sort, *ρ*_*c*_ should also go down with increasing magnetic field, but stay temperature independent because of scattering on domain walls.

In summary, the MM transition in Sr_4_Ru_3_O_10_ as a function of magnetic field and rotation angle between *H* ⊥ *c* and *H* || *c* has been studied in great detail by magnetization measurements down to lowest temperatures in a SQUID magnetometer. Our experimental results reveal a reduced magnetic moment in the plane of rotation which was never recognized before. It is robust to (*ab*) in-plane rotation. We find furthermore that the double step at the MM transition is stable down to lowest temperatures of 0.46 K. Our experimental results are interpreted in a strict localized picture with magnetic moments of different sizes sitting on inner and outer RuO_6_ layers in the crystal structure. We completed our study with numerical calculations based on energy minimization including Zeeman effect, magnetocrystalline anisotropy and antisymmetric exchange and compared them with our experimental data. We conclude that all three contributions are essential ingredients to understand the behavior of the magnetization and that a *Dzyaloshinsky* - *Moriya* like component is crucial to model a reduced magnetic moment in Sr_4_Ru_3_O_10_.

## Methods

Sr_4_Ru_3_O_10_ single crystals were grown in an image furnace by a floating zone technique^40^ and characterized by energy dispersive spectroscopy, scanning electron microscopy, electron backscattering diffraction and x-ray diffraction techniques.

Magnetization measurements in fields up to 7 T were carried out in a Quantum Design MPMS SQUID magnetometer equipped with a standard ^4^He setup for measurements between 1.7 K and 100 K and in an iQuantum ^3^He insert that fits inside the MPMS sample space for the temperature range 0.46 K to 2 K. Excellent agreement between the ^3^He and ^4^He data was observed in the temperature range of overlap. Angular dependent measurements at 1.8 K were obtained with a mechanical rotator mounted inside the MPMS magnetometer. Note that the MPMS operates with a pair of coils for signal detection mounted parallel (longitudinal coil) and perpendicular (transversal coil) to the applied magnetic field. The rotator is aligned with the rotation axis normal to the plane defined by both SQUID coil axes.

The measured single crystal has a rectangular shape of (1.87 × 2.99) mm^2^ in *ab* and 0.54 mm along the crystallographic *c*-direction. We considered demagnetization effects by approximating the sample shape with an ellipsoid^41^ and estimated small correction fields of −30 mT for *H* ⊥ *c* and −140 mT for *H* || *c*, which are negligible for the analysis and discussion of our magnetization results close to the metamagnetic transition.
